# CLE peptide signaling in plant-microbe interactions

**DOI:** 10.3389/fpls.2024.1481650

**Published:** 2024-10-23

**Authors:** Satoru Nakagami, Taiki Kajiwara, Kenichi Tsuda, Shinichiro Sawa

**Affiliations:** ^1^ National Key Laboratory of Agricultural Microbiology, Hubei Hongshan Laboratory, Hubei Key Laboratory of Plant Pathology, College of Plant Science and Technology, Huazhong Agricultural University, Wuhan, China; ^2^ Shenzhen Institute of Nutrition and Health, Huazhong Agricultural University, Wuhan, China; ^3^ Shenzhen Branch, Guangdong Laboratory of Lingnan Modern Agriculture, Genome Analysis Laboratory of the Ministry of Agriculture and Rural Affairs, Agricultural Genomics Institute at Shenzhen, Chinese Academy of Agricultural Sciences, Shenzhen, Guangdong, China; ^4^ Graduate School of Science and Technology, Kumamoto University, Kumamoto, Japan; ^5^ International Research Center for Agricultural and Environmental Biology, Kumamoto University, Kumamoto, Japan; ^6^ International Research Organization for Advanced Science and Technology (IROAST), Kumamoto University, Kumamoto, Japan; ^7^ Institute of Industrial Nanomaterial (IINA), Kumamoto University, Kumamoto, Japan

**Keywords:** peptide, plant-microbe interaction, systemic signaling, plant immunity, nodulation, phytoparasitic nematode, AM symbiosis

## Abstract

Cell-cell communication is essential for both unicellular and multicellular organisms. Secreted peptides that act as diffusive ligands are utilized by eukaryotic organisms to transduce information between cells to coordinate developmental and physiological processes. In plants, The *CLAVATA3/EMBRYO SURROUNDING REGION-RELATED* (*CLE*) genes encode a family of secreted small peptides which play pivotal roles in stem cell homeostasis in various types of meristems. Accumulated evidence has revealed that CLE peptides mediate trans-kingdom interactions between plants and microbes, including pathogens and symbionts. This review highlights the emerging roles of CLE peptide signaling in plant-microbe interactions, focusing on their involvement in nodulation, immunity, and symbiosis with arbuscular mycorrhizal fungi. Understanding these interactions provides insights into the sophisticated regulatory networks to balance plant growth and defense, enhancing our knowledge of plant biology and potential agricultural applications.

## 1 Introduction

**Figure 1:**
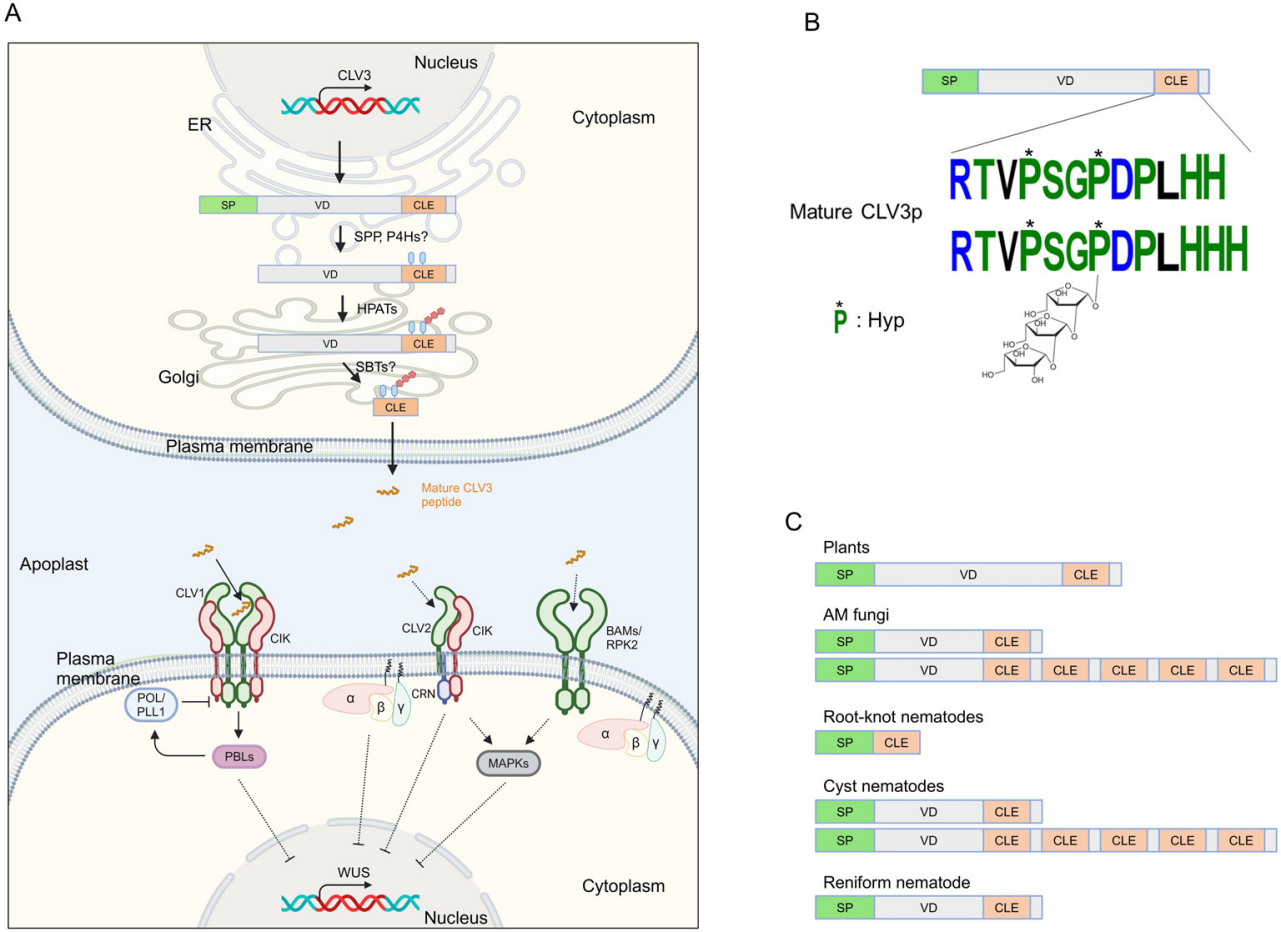
Illustrating signaling components of the CLAVATA pathway. **(A)** CLV3 signaling in the shoot apical meristem. The CLV3 prepropeptide is processed by hydroxyproline (Hyp) O-arabinosyltransferases (HPATs), and presumably by a signal peptide peptidase (SPP), prolyl-4-hydroxylases (P4Hs) and subtilases (SBTs) in ER-Golgi, and then mature CLV3 peptide is secreted to the apoplast. The CLV3 peptide is recognized by plasma membrane-associated receptor complexes, which in turn, WUSCHEL (WUS) is repressed through heterotrimeric G proteins, receptor-like cytoplasmic kinases (RLCKs) PBS1-LIKEs (PBLs), phosphatases POLTERGEIST (POL) and POL-LIE 1 (PLL1), and MAPKs. Dashed lines indicate putative or indirect pathways. **(B)** A model of the CLV3 prepropeptide and CLV3 peptide. The CLV3 prepropeptide consists of the N-terminal signal peptide (SP), the C-terminal CLE domain, and the variable domain (VD) between SP and CLE. **(C)** Representative structures of CLE prepropeptides produced by plants and microbes. Figure adapted from images created with BioRender.com.

Cell-to-cell communication is essential for multicellular organisms to coordinate their growth and development. Plants transmit information between cells through phytohormones, proteins/peptides, small RNAs, and metabolites. Secreted peptides acting as ligands are defined as extracellular proteins (less than 100 amino acids in length) derived from precursor proteins called prepropeptides (Tavormina et al., 2015). Peptide ligands are typically recognized by receptor kinases localized at the plasma membrane, thereby provoking an intracellular signal transduction cascade that changes the activity of downstream genes to modify cellular programs. The CLAVATA3 (CLV3)/EMBRYO SURROUNDING REGION-RELATED (CLE) gene family is one of the largest signaling peptide families in plants, with model plant *Arabidopsis thaliana* (hereafter Arabidopsis) genome containing 32 *CLE* genes. Canonical CLE prepropeptides possess a signal peptide at the N-terminus, a highly conserved motif at the C-terminus called the CLE domain, and a variable domain between the signal peptide and the CLE domain (Betsuyaku et al., 2011) (**Figure 1**). It is thought that CLE prepropeptides are cleaved by an endoplasmic reticulum-localized signal peptide peptidase to remove their signal peptide, resulting in a propeptide (**Figure 1A**). Propeptides are processed by post-translational modifications and are proteolytically cleaved at the N- and C- termini of the CLE domain by subtilases to generate a mature peptide in length of 12-14 amino acids (Betsuyaku et al., 2011; Stührwohldt et al., 2020). For instance, the mature CLV3 peptide has been identified as two distinct forms; (1) a 12 amino acids peptide with hydroxyproline (Hyp) residues in the 4th and 7th positions; (2) a 13 amino acids peptide that, with Hyp at the 4th position and tri-arabinosylated Hyp at the 7th position, contains an additional histidine residue at the 13th position (**Figure 1B**), those two forms can directly bind to extracellular domain of the plasma membrane-localized leucine-rich repeat receptor-like kinase (LRR-RLK) AtCLV1 (Kondo et al., 2006; Ohyama et al., 2009). The tri-arabinosylation is catalyzed by the Hyp O-arabinosyltransferases (HPATs) localized at Golgi (Ogawa-Ohnishi et al., 2013). This modification is critical for the binding affinity with CLV1, and therefore important for the bioactivity of the CLV3 peptide.

Secreted mature CLE peptides are perceived by LRR-RLKs and coreceptors such as CLV1 and CLV3 INSENSITIVE RECEPTOR KINASEs (CIKs), LRR receptor-like protein (LRR-RLP) CLV2, and RLK CORYNE (CRN), which lacks the extracellular domain and acts with CLV2 and CIKs (Hu et al., 2018; Fletcher, 2020; Jones et al., 2021) (**Figure 1A**). CLV-type receptor complexes have been well characterized for their role in shoot apical meristem (SAM) maintenance, where CLE peptides are recognized by their cognate receptors, reading to the activation of an intracellular signaling cascade such as the receptor-like cytoplasmic kinases (RLCKs) PBS1-LIKEs (PBLs), the protein phosphatases POLTERGEIST (POL) and POLTERGEIST-LIKE1 (PLL1), MAP kinases, and the heterotrimeric GTP binding proteins (Yamaguchi et al., 2016; Wang et al., 2021c). In the SAM, the expression of the homeodomain transcription factor *WUSCHEL* (*WUS*) that promotes stem cell activity is suppressed by the CLV3-receptors module, thereby maintaining stem cell homeostasis. CLE-receptor modules also regulate stem cell homeostasis in inflorescence meristem, root apical meristem, and vascular meristem, and some physiological responses (Fletcher, 2020; Bashyal et al., 2023).

In nature, plants are associated with various microbes including bacteria, fungi, oomycetes, viruses, and nematodes. Plant-associated microbes affect host developmental processes and physiological responses as infection outcomes; in turn, host plants regulate microbial associations and behaviors to shape optimal interactions between plants and microbes. Accumulating evidence has depicted the emerging roles of CLE signaling in plant-microbe interactions. In this review, we describe CLE signaling pathways participating in plant-microbe interaction regulation and how CLE peptides and their receptors may respond to biotic and abiotic stimuli and culminate in changes to downstream signal transduction.

## 2 Regulation of nodule formation by host CLE signaling

**Figure 2:**
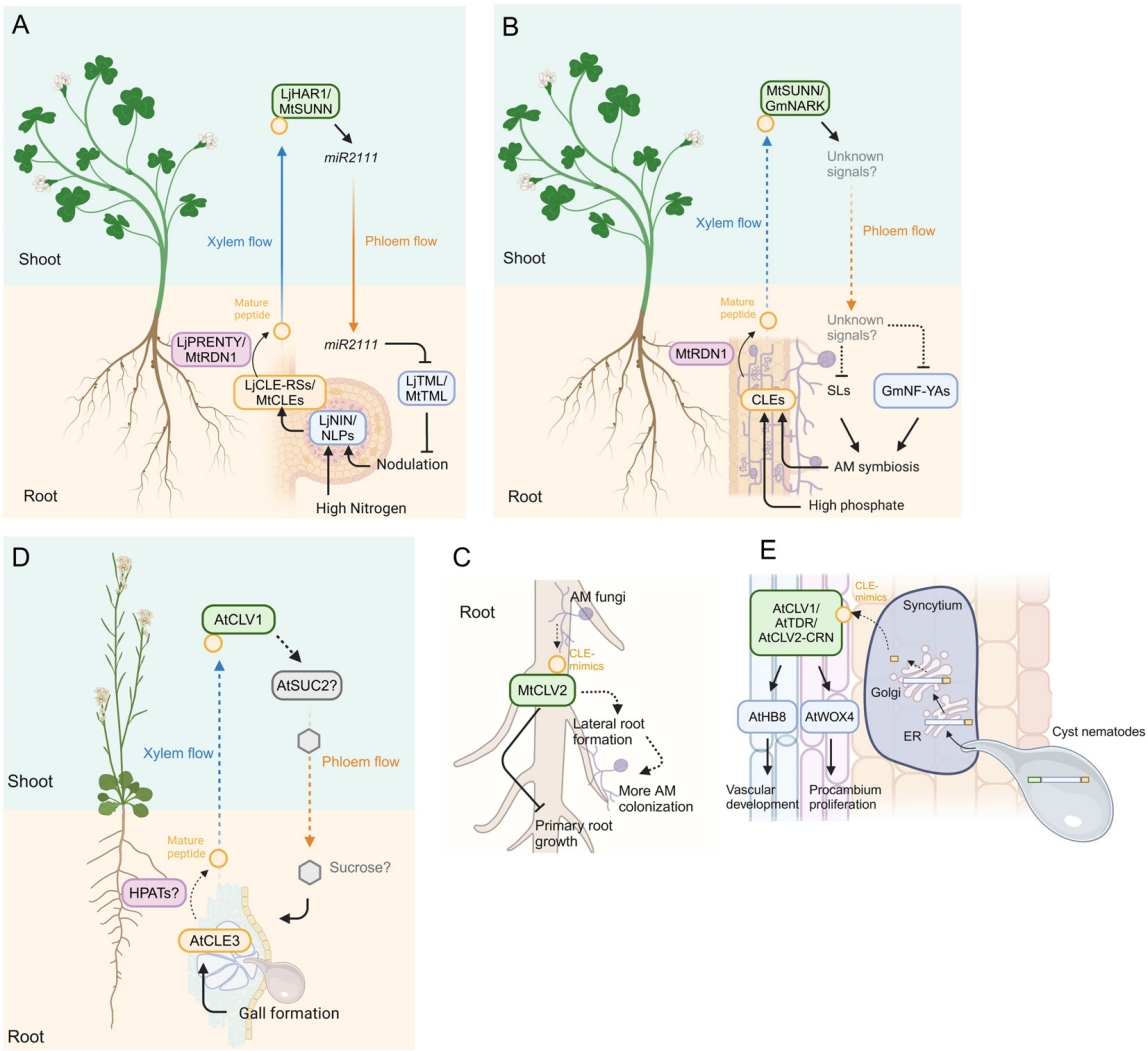
Model of CLE signaling in plant-microbe interactions. **(A)** Model of systemic signals mediated by CLE signaling in legume-rhizobium interactions. The transcription factors NODULE INCEPTION (NIN) and NIN-LIKE PROTEINs (NLPs) induce *CLE* gene expression in response to nodulation and high nitrogen conditions, respectively. Mature CLE peptides are post-translationally modified by HPATs LjPRENTY or MtRDN1 and translocate to the shoot via xylem, where the cognate receptors recognize the mature peptides. The *miR2111* translocates from the shoot to the root via phloem and suppresses excessive nodulation by downregulating *TOO MUCH LOVEs* (*TMLs*) expression. **(B)** Model of systemic signals mediated by CLE signaling in plant-AM fungi interactions. AM colonization and high phosphate conditions induce the accumulation of CLE peptides, which are post-translationally modified by RDN1 and translocated to the shoot, where the cognate receptors recognize the mature CLE peptides. Strigolactones (SLs) and NUCLEAR FACTOR-YA transcription factors (NF-YAs) accumulation is suppressed through yet unknown long-distance signals translocating from the shoot to the root downstream of the CLE peptides perception, thereby suppressing additional AM symbiosis. **(C)** Model of local signal mediated by CLE peptide mimics in *M. truncatula*. AM fungi produce CLE mimics that inhibit primary root growth partially dependent on CLV2. The AM-CLE mimics also induce more AM colonization probably due to their induction effect of lateral root formation. **(D)** Model of systemic signal mediated by CLE signaling in plant and root-knot nematode interaction in Arabidopsis. The *CLE3*, which is upregulated in root-knot nematode-induced galls, regulates gall formation in a shoot-expressed CLV1-dependent manner, presumably via SUCROSE-PROTON SYMTORTER 2 (SUC2)-regulated sucrose translocation from the shoot to the root. **(E)** Model of local signal mediated by CLE peptide mimics in plant-cyst nematode interaction in Arabidopsis. Mature CLE mimics are secreted to the apoplast via the host ER-Golgi network. The host receptors recognize the mimics and induce expression of *WUSCHEL-RELATED HOMEOBOX 4* (*WOX4*) and *HOMEOBOX GENE 8* (*AtHB8*) which regulate procambium activity and vascular formation, thereby supporting cynsytium formation. Dashed lines indicate putative or indirect pathways. Figure adapted from images created with BioRender.com.

The relationship between legume plants (members of the family Fabaceae) and nitrogen-fixing bacteria, so-called rhizobia, is one of the most successful symbioses in nature. Endosymbiotic rhizobia inhabiting nodules which are lateral organs formed on the legume roots supply ammonia converted from atmospheric nitrogen to the host, while the host supplies malate converted from sucrose as the primary source of carbon (Udvardi and Poole, 2013). Excessive nodule formation can be detrimental to the host growth since plants lose carbon sources assimilated by photosynthesis. Therefore, legume plants have evolved a sophisticated regulatory mechanism called autoregulation of nodulation (AON) to prevent excess nodulation (Chaulagain and Frugoli, 2021). AON is a complex mechanism modulated through long-distance signaling between roots and shoots and is highly conserved across legume plants (Chaulagain and Frugoli, 2021; Li et al., 2022). Here, we focus on the role of signaling components constituted by CLE peptides and their cognate receptors mainly in *Lotus japonicus* and *Medicago truncatula* (**Figure 2A**).

CLE peptides act as root-derived mobile signals in AON. In *L. japonicus*, a model legume plant, the expression of *LjCLE-ROOT SIGNAL 1* (*LjCLE-RS1*), *LjCLE-RS2*, and *LjCLE-RS3* is induced in the roots where rhizobia infect (Okamoto et al., 2009; Nishida et al., 2016). Experiments using transgenic hairy roots revealed that constitutive expression of these genes suppressed nodulation on not only transgenic hairy roots but also untransformed roots (Okamoto et al., 2009; Nishida et al., 2016). The mature LjCLE-RS2 peptide was detected in xylem sap collected at the hypocotyl of the plants that have transgenic hairy roots transformed with *LjCLE-RS2*, indicating that LjCLE-RS2 peptide is a root-derived mobile signal (Okamoto et al., 2013). As well as the mature form of AtCLV3 peptide, mature LjCLE-RS2 peptide is a 13 amino acids peptide that is derived from the C-terminal region of the precursor polypeptide and the 7th Hyp residue of this peptide is modified with three residues of arabinose (Ohyama et al., 2009; Okamoto et al., 2013). These *CLE* genes that act as a negative regulator on AON are evolutionarily conserved in other legumes, such as *M. truncatula*, *Glycine max* (soybean), *Pisum sativum* (pea), and *Phaseolus vulgaris* (common bean). It has been shown that *CLE* genes of *M. truncatula* (*MtCLE12*, *MtCLE13*, *MtCLE34*, and *MtCLE35*), *G. max* (*RHIZOBIA-INDUCED CLE 1*: *GmRIC1* and *GmRIC2*), *P. sativum* (*PsCLE12*, *PsCLE13*, and other ten *PsCLEs*) and *P. vulgaris* (*PvRIC1* and *PvRIC2*) are upregulated by rhizobial infection (Mortier et al., 2010; Lim et al., 2011; Reid et al., 2011; Ferguson et al., 2014; Alves‐Carvalho et al., 2015; Kassaw et al., 2017; Samorodova et al., 2018; Lebedeva et al., 2020; Mens et al., 2021). Constitutive expression of these genes in transgenic hairy roots leads to suppression of nodulation on systemic roots. However, the mature forms of most of these CLE peptides have yet to be elucidated. In *L. japonicus*, *M. truncatula*, and *G. max*, upregulation of *CLE* genes (*LjCLE-RS1*, *LjCLE-RS2*, *MtCLE13*, *GmRIC1*, and *GmRIC2*) in AON requires NODULE INCEPTION (NIN), which is an indispensable transcription factor for multiple aspects of rhizobial symbioses and is consistently lost in non-nodulating species (Soyano et al., 2014; Wang et al., 2019; Laffont et al., 2020; Shen and Feng, 2024). NIN has evolved from NIN-LIKE PROTEINs (NLPs) which control the expression of nitrate-responsive genes (Konishi and Yanagisawa, 2013; Marchive et al., 2013), suggesting that the regulation of CLE gene expression on AON may have evolved from the mechanism for fine-tuning in planta nitrogen status.

Post-translational modification of CLE peptides is critical for AON. An *M. truncatula* mutant lacking *ROOT DETERMINED NODULATION 1* (*MtRDN1*) gene that encodes HPAT exhibits hyper nodulation phenotype (Schnabel et al., 2011; Kassaw et al., 2017). Through a combination of biochemical and genetic analyses to test functional implications of arabinosylation on AON, Imin and colleagues demonstrated that chemically synthesized MtCLE12 and MtCLE13 peptides with tri-arabinosylation at the 7th Hyp residue exerted nodulation inhibition in the wild-type and *rdn1* mutant when they were applied to the roots or the cotyledons, but these oligopeptides without this modification no longer inhibited nodulation (Imin et al., 2018). Consistent with this, tri-arabinosylated LjCLE-RS1/2/3 and GmRIC1/2 peptides can weaken the hyper nodulation phenotype of mutants lacking LjPLENTY and PsNOD3, which are orthologs to MtRDN1 (Hastwell et al., 2019; Yoro et al., 2019). The MtRDN1 and LjPLENTY are localized to the Golgi (Kassaw et al., 2017; Yoro et al., 2019). Indeed, tri-arabinosylated LjCLE-RS2 peptide was detected in shoot xylem sap collected from soybean plants that developed transformed hairy roots having the genomic region of *LjCLE-RS2*; its modification significantly impacts binding affinity to the receptor mentioned below (Okamoto et al., 2013). These observations indicate that HPATs-mediated tri-arabinosylation of CLE peptides plays a key role in AON. As Kassaw and coauthors have provided evidence that MtRDN1 is required for the function of MtCLE12, but not MtCLE13 (Kassaw et al., 2017), further studies would reveal substrate preferences/specificities of HPATs for individual CLE peptides in each legume.

CLE-mediated AON requires shoot-acting cognate receptors. Grafting experiments and genetic assays showed that the LjCLE-RS peptides were recognized by shoot-acting LRR-RLKs HYPERNODULATION ABERRANT ROOT FORMATION 1 (LjHAR1) and KLAVIER (LjKLV) and LRR-RLP LjCLV2, which have high similarity to AtCLV1, RECEPTOR-LIKE PROTEIN KINASE 2 (AtRPK2) and AtCLV2, respectively (Wopereis et al., 2000; Krusell et al., 2002, 2011; Nishimura et al., 2002; Oka-Kira et al., 2005; Okamoto et al., 2009, 2013; Miyazawa et al., 2010; Nishida et al., 2016). Indeed, synthetic tri-arabinosylated LjCLE-RS1 and -RS2 peptides directly bind to the extracellular domain of LjHAR1 (Okamoto et al., 2013). Akin to AtCLV1, LjHAR1, LjKLV, and LjCLV2 can form isoforms, with homo- and hetero-dimerization (Miyazawa et al., 2010; Krusell et al., 2011). Since AtCLV2 acts with RLK AtCRN through a protein-protein interaction manner, LjCLV2 may also heterodimerize with an unidentified *L. japonicus* CRN orthologue (Bleckmann et al., 2009; Krusell et al., 2011). AtCLV1 and AtCLV2 like receptors have been characterized as negative regulators of nodulation in other legume plants: SUPER NUMERIC NODULES (MtSUNN, an AtCLV1 paralog) and MtCLV2 in *M. truncatula*, NODULE AUTOREGULATION RECEPTOR KINASE (GmNARK/PvNARK, an AtCLV1 paralog) in *G. max* and *P. vulgaris*, and PsSYM29 and PsSYM28 (AtCLV1 and AtCLV2 paralog, respectively) in *P. sativum* (Krusell et al., 2002, 2011; Searle et al., 2003; Schnabel et al., 2005; Ferguson et al., 2014). In *M. truncatula*, MtCLV2 and MtCRN can form heteromers, in heterologous expression system in tobacco leaf cells, and they act as a negative regulator on AON downstream of MtCLE12 and MtCLE13 peptides (Crook et al., 2016; Nowak et al., 2019). Interestingly, unlike *AtCLV1*, a mutant lacking *LjHAR1* does not show any shoot phenotypes, whereas *klv* mutant of *L. japonicus* shows *clv1*-like shoot phenotypes such as fasciated stems, suggesting that AtCLV1 orthologs of legumes have evolved to control nodulation in shoots (Oka-Kira et al., 2005; Miyazawa et al., 2010).

CLE-mediated regulation of nodule formation is exerted by not only rhizobial infection but also several abiotic conditions. For instance, *LjCLE-RS2*, *LjCLE-RS3*, *LjCLE40*, *MtCLE34*, *MtCLE35*, and *NITRATE-INDUCED CLE* (*GmNIC1*) are induced in response to high nitrogen conditions, and among them, overexpression of *LjCLE-RS2*, *LjCLE-RS3*, *MtCLE35*, and *GmNIC1* suppresses nodulation through their cognate receptors (Okamoto et al., 2009; Reid et al., 2011; Nishida et al., 2016; Lebedeva et al., 2020; Lebedeva et al. 2022; Mens et al., 2021; Moreau et al., 2021). Recent studies have shown that LjNLP1, LjNLP4, MtNLP1, GmNLP1, and GmNLP4, which are homologs of NIN transcription factors, are required to induce these nitrogen-responsive *CLEs* by directly binding to their promoter regions (Luo et al., 2021; Moreau et al., 2021; Nishida et al., 2021; Fu et al., 2024). Phosphate is another abiotic factor that controls nodulation mediated by CLE signaling. *PvRIC1* and *PvRIC2* are upregulated under low phosphate conditions as well as rhizobial infection (Isidra-Arellano et al., 2018; Isidra‐Arellano et al., 2020). Suppression of nodulation by low phosphate is not observed in the *NARK* mutants of *G. max* and *P. vulgaris*, suggesting that the peptides-receptors module required for phosphate-responsive nodule regulation is shared with AON pathway.

As described above, root-derived CLE peptides travel to the shoot where they are perceived by shoot-acting receptor complexes, and, in turn, downstream signals suppress excessive nodule formation in roots. Therefore, there should be the signaling molecule(s) that, downstream of CLE perception, translocates from shoots to roots. To regulate nodule numbers during rhizobial infection, two shoot-to-root mobile signals have been proposed by studies of *L. japonicus* and *M. truncatula* to date: (1) phytohormone cytokinin and (2) micro-RNA *miR2111* (Sasaki et al., 2014; Tsikou et al., 2018; Gautrat et al., 2020; Okuma et al., 2020; Zhang et al., 2021). Rhizobial infection activates the cytokinin production in the shoot by inducing a cytokinin biosynthesis gene *LjIPT3* in a LjHAR1-dependent manner, which can suppress nodule formation (Sasaki et al., 2014). An experiment using isotope-labeled cytokinin demonstrated that cytokinin fed to leaves was transported to roots in *L. japonicus*, probably via phloem, suggesting cytokinin is a shoot-to-root mobile signal downstream of LjHAR1 on AON. On the contrary to cytokinin, an *miR2111* has been characterized as a positive regulator of nodulation. Mature *miR2111*s were highly accumulated in uninfected plants to repress nodule suppressor *TOO MUCH LOVE* (*TML*) in roots. In contrast, the expression level of *miR2111s* in leaf phloem was downregulated depending on the signaling module constituted by LjCLE-RS peptides and LjHAR1 in infected plants, thereby derepressing *TML* to inhibit excessive nodulation (Tsikou et al., 2018; Okuma et al., 2020). The *miR2111* expression in shoots was required for *TML* repression in roots, suggesting *miR2111* acts as a shoot-to-root mobile signal on AON. This *miR2111*-mediated systemic AON through the CLE signaling is conserved in *M. truncatula* and *G. max* as well (Gautrat et al., 2020; Moreau et al., 2021; Zhang et al., 2021).

Collectively, these conserved peptide-receptor pairs of legumes function in AON during rhizobial colonization in the same context. Interestingly, some parts of these signaling components are often utilized for regulation of lateral organ formations such as lateral root formation in both leguminous and non-leguminous plants (Araya et al., 2014; Huault et al., 2014; Soyano et al., 2019; Zhang et al., 2021; Hayashi-Tsugane and Kawaguchi, 2022; Kang et al., 2022; Lebedeva et al., 2023; Nakagami et al., 2023a; Sexauer et al., 2023). Therefore, it seems that organogenesis-regulating molecular network mediated by CLE signaling shares with the lateral organ developmental pathway, which is regulated in response to changing nutritional status. Nevertheless, our knowledge of CLE signaling components on AON is still insufficient, necessitating further investigation.

## 3 Regulation of fungal colonization by CLE signaling

The interaction between plants and Arbuscular Mycorrhizal (AM) fungi that belong to the phylum Glomeromycotina is often recognized as mutualistic because host plants receive phosphorus and some other micronutrients from AM fungi, with the hosts providing carbohydrates and lipids to the fungus (Shi et al., 2023). The host plants suppress excessive AM colonization to prevent excess loss of the photosynthetic products. Similar to AON, the hosts exert a long-distance negative feedback regulation that controls AM symbiosis in already colonized roots, called autoregulation of mycorrhization (AOM). Here, we introduce a molecular mechanism involved in AOM mediated by the CLE signaling pathway (**Figure 2B**).

AM colonization induces *CLE* genes in roots of *L. japonicus* (*LjCLE7* and other 5 *LjCLEs*), *M. truncatula* (*MtCLE53*), *Solanum lycopersicum* (tomato) (*SlCLE11*), and a monocotyledon *Brachypodium distachyon* (*Bd1g49027* and *Bd2g50170*) (Handa et al., 2015; Müller et al., 2019; Karlo et al., 2020; Wulf et al., 2023). *M. truncatula* plants ectopically overexpressing *MtCLE53* in the roots showed low colonization levels of the AM fungus, while a mutant lacking *MtCLE53* was more colonized than the wild-type plant (Müller et al., 2019; Karlo et al., 2020). Overexpression of *MtCLE33*, which is not induced by AM colonization but phosphate-inducible, also reduced AM colonization, suggesting the CLE signaling negatively controls AM symbiosis responding to both AM colonization and the nutritional status (Müller et al., 2019). In *S. lycopersicum* plants, loss- and gain-of-function studies showed that *SlCLE11* repressed AM symbiosis by responding to AM infection (Wulf et al., 2023). Akin to the function of the CLE peptides on AON, genes encoding HPATs are required for the function of the CLEs involving AOM. The mutant lacking *MtRDN1* showed high levels of AM colonization, with overexpression of *MtCLE53* in the roots showing no change in AM colonization in the *rdn1*, suggesting that MtCLE53 requires HPAT for AOM (Karlo et al., 2020). Corresponding with the effect of HPAT on the CLE function in *M. truncatula*, a defect of *FASCIATED INFLORESCENCE* (*SlFIN*), which encodes HPAT in *S. lycopersicum*, caused an increase in AM colonization levels and lost the low colonization phenotype caused by *SlCLE11* overexpression (Wang et al., 2021a; Wulf et al., 2023). Although genetic studies have revealed that the *HPATs* are required for *MtCLE53* and *SlCLE11* function, whether mature forms of these peptides are indeed arabinosylated has not been identified yet. Furthermore, how *CLE* genes are induced during AM symbioses is still elusive, which necessitates further investigation.

Recognition of CLE peptides involved in AOM requires its cognate receptors, and accumulating evidence indicates that orthologs of *AtCLV1* or *AtCLV2* are signaling components of AOM. It has been shown that mutants defective in *LjHAR1*, *MtSUNN*, *GmNARK*, *PsNARK*, *SlCLV2*, *FASCIATED AND BRANCHED* (*SlFAB*), and *FLORAL ORGAN NUMBER 1* (*BdFON1*) exhibit more colonization phenotypes (Morandi et al., 2000; Zakaria Solaiman et al., 2000; Meixner et al., 2005; Müller et al., 2019; Karlo et al., 2020; Wang et al., 2021a). The suppressive effect of *MtCLE53* overexpression on AM symbiosis is MtSUNN dependent, suggesting MtSUNN is a cognate receptor for MtCLE53p (Karlo et al., 2020); however, a receptor required for MtCLE33 peptide recognition in AOM has not been characterized. Considering the observation that *MtCLE53* and *MtCLE33* are expressed in the root vascular tissues and MtSUNN is expressed in the vasculatures of both the root and shoot (Schnabel et al., 2012; Müller et al., 2019), the MtCLEs-MtSUNN module could act locally and/or systemically on AOM. Further investigations using grafting and split-root system would be valuable in distinguishing between these possibilities. Grafting experiments showed that shoot-expressed GmNARK controlled AM colonization systemically (Sakamoto and Nohara, 2009). Genetic studies revealed that SlCLE11 required neither SlFAB nor SlCLV2 (Wulf et al., 2023), suggesting the possibility that there are other CLE peptides involved in AOM and other receptor(s) that can perceive SlCLE11. Grafting experiments showed that SlFAB acts only in roots, while SlCLV2 acts in both roots and shoots (Wang et al., 2021a); whether regulation of AOM mediated by distinct CLE-receptor modules is exerted locally or systemically remains unclear.

Several nutritional conditions also trigger CLE-mediated regulation of AM symbiosis. As described above, *MtCLE33* is not induced by AM colonization but phosphate-inducible, with *MtCLE33* overexpression reducing AM colonization in a MtSUNN-dependent manner (Müller et al., 2019). Similarly, *SlCLE10* is upregulated in responding to high nitrogen and phosphate, and *SlCLE10* overexpression suppresses AM colonization (Wulf et al., 2023); however, its cognate receptor has not been identified yet. Although other *CLE* genes (two *LjCLEs*, four *MtCLEs*, three *SlCLEs*, and three *BdCLEs*) that respond to phosphate and some other nutrition status have been characterized in several plant species (Funayama-Noguchi et al., 2011; Handa et al., 2015; Müller et al., 2019; Karlo et al., 2020; Wulf et al., 2023), there is a lack of evidence as to whether these *CLEs* control AM symbioses.

Following CLE perception by their receptor modules, its downstream signaling regulates AM colonization in roots. Gene expression analyses revealed that biosynthesis genes of phytohormone strigolactones that promote AM colonization were downregulated both in *MtCLE53* and *MtCLE33* overexpressing roots in an MtSUNN-dependent manner, in consequence, strigolactones content was reduced in the roots (Müller et al., 2019). Exogenous treatment of a strigolactone analog GR24 to the *MtCLEs* overexpressing roots rendered AM colonization levels, indicating that strigolactone signaling is downstream of the MtCLE-MtSUNN module on AM symbiosis. Other factors downstream of the CLE-receptor module are transcription factor *NUCLEAR FACTOR-Y* (*NF-Y*) genes. Gene expression studies using split-root experiments showed that *GmNF-YA1a* and *GmNF-YA1b* were systemically downregulated in non-infected roots GmNARK-dependently, with knocking down of these two genes weakening hyper AM colonization phenotype of *nark* mutants (Schaarschmidt et al., 2013). Considering that the CLE peptides are perceived by their cognate receptors in the shoot during AM symbiosis, a shoot-derived descending signal is required for the downstream factors acting in the roots. Further investigation would identify yet unknown shoot-to-root factors. Previous studies have identified two shoot-to-root mobile factors involved in AM symbiosis regulation, ELONGATED HYPOCOTYL 5 (HY5) transcription factor that regulates strigolactone levels in roots, and micro RNAs such as *miR399* that induces phosphate transporter genes in response to phosphate starvation (Wu et al., 2013; Müller and Harrison, 2019; Ge et al., 2022). It would be interesting to investigate whether these mobile factors are downstream of CLE signaling.

Plant-associated microbes also produce phytohormones or their mimics that manipulate phytohormone signaling networks in plants, thereby supporting their colonization of the hosts (Nakano et al., 2022). Le Marquer et al. (2019) identified *CLE*-like genes in the genome of five AM species. The prepropeptides of four *Rhizophagus* species were comprised of a predicted signal peptide at the N-terminus, a relatively shorter variable domain, and a CLE domain at the C-terminus as well as that of plants, while that of *Gigaspora rosea* possessed five CLE domains (**Figure 1C**). Expression of the fungal *CLE*-like genes of *Rhizophagus irregularis* and *G. rosea* (*RiCLE1* and *GrCLE1*) was induced during symbiotic conditions. Exogenous application of synthetic RiCLE1 peptide on roots of *M. truncatula*, *P. sativum* and *A. thaliana* reduced primary root growth (Le Marquer et al., 2019). Consistent with a previous study in AtCLE peptides, this effect was partially dependent on CLV2 type receptor but not CLV1 (Fiers et al., 2005; Le Marquer et al., 2019), suggesting that RiCLE1 peptide is perceived by the host receptors. In addition, RiCLE1 peptide application to *M. truncatula* roots increased lateral root branching and AM colonization (Le Marquer et al., 2019). Since AM colonization often leads to an increase in lateral root formation and lateral roots are preferentially colonized by AM fungi (Sukumar et al., 2013), these observations raise the possibility that their increased abundance mediated by the fungal CLE peptides may lead to an enlarged interface for the plant-AM fungi interaction (**Figure 2C**). However, there is the possibility that increasing lateral root growth may merely be caused by the inhibition of primary root growth (Potters et al., 2009), further studies should investigate the biological implications of the fungal CLE peptides on AM symbiosis.

## 4 Roles of CLE signaling in plant-nematode interactions

Phytoparasitic nematodes are obligate parasitic animals, which are recognized to cause severe economic losses in agriculture worldwide (Jones et al., 2013). Accumulating evidence has shown that the CLE signaling pathway is utilized for successful infection. Here, we introduce the roles of CLE signaling in plant-nematode interactions.

### 4.1 Root-knot nematodes

Root-knot nematodes (RKN; *Meloidogyne* spp.) parasite most vascular plants and are distributed worldwide. Infective juveniles move toward plant roots in the soil and invade through the root meristematic zone. After invasion, they inject an effector cocktail into the host procambial cells that suppresses host defense responses and manipulates host developmental pathways, thereby triggering the formation of their feeding sites called galls or root-knots on the host root by modulating the host auxin pathway (Favery et al., 2016; Olmo et al., 2020; Suzuki et al., 2022; Abril-Urias et al., 2023; Noureddine et al., 2023). Gene expression analyses in *M. incognita*-infected Arabidopsis showed that *AtCLE1*, *AtCLE3*, *AtCLE4*, and *AtCLE7*, which are orthologs of the symbiosis-induced *CLEs* described above, were upregulated in galls (Yamaguchi et al., 2017; Nakagami et al., 2023b). Single mutants lacking *AtCLE1*, *AtCLE3*, or *AtCLE7* exhibited reduced gall numbers, and the higher-order mutant of *AtCLE1* to *AtCLE7* (*cle1~7*) showed an additive effect on the reduction of gall formation, whereas CLE overexpression led to increased gall formation, showing that these *CLE* genes positively regulate gall formation (Nakagami et al., 2023b). Grafting and split-root experiments revealed that the CLE-mediated regulation of gall formation required shoot-expressed AtCLV1, with synthetic AtCLE3 peptide directly binding to the extracellular domain of AtCLV1, indicating that AtCLE1~7-AtCLV1 module is the systemic pathway. Consistent with this, MtSUNN positively controls gall formation during *M. javanica* infection (Costa et al., 2020). These results raise the question of how shoot-expressed AtCLV1 regulates gall formation on roots after AtCLE peptides perception. A recent study showed that *AtCLE2* and *AtCLE3* were induced in roots when the roots were exposed to sucrose starvation (Okamoto et al., 2022). Root-expressed *AtCLE1*~*AtCLE7* maintained *SUCROSE-PROTON SYMTORTER 2* (*SUC2*) expression in the leaves, thereby balancing the root sucrose levels and growth. Indeed, exogenous sucrose application to the *M. incognita*-infected roots compensated the gall-reducing phenotype of the *cle1~7* and *clv1* mutants (Nakagami et al., 2023b), suggesting that maintenance of sucrose levels in roots mediated by leaf-expressed SUC2 is a possible downstream mechanism of AtCLE peptides/CLV1 on RKN infection (**Figure 2D**). Therefore, *M. incognita* may manipulate host sucrose homeostasis to acquire sucrose as a nutrient efficiently and/or allocate sucrose as an energy source for gall formation.

Our knowledge of AtCLE/CLV1 on plant-nematode interactions is still fragmented. For instance, there is a lack of explanation for how *M. incognita* induces these *AtCLEs* in the host roots, whether these AtCLEs require arabinosylation for their function, and if so, which HPAT arabinosylates these AtCLEs. In another aspect of CLE signaling in plant-RKN interactions, like cyst nematodes (see below), *M. incognita* possesses a gene harboring a ligand-like motif somewhat similar to the CLE motif, named *16D10*, that is required for RKN parasitism, and the 16D10 peptide can interact with host transcription factors, not receptor-like proteins, making it unique among secreted peptides (Huang et al., 2006b, 2006a; Yang et al., 2013). In addition, RKNs harbor *CLE*-like genes constituted by only a signal peptide domain and a CLE domain, but their function is unknown (Mitchum and Liu, 2022) (**Figure 1C**). However, several studies provide evidence that RKNs possess functional peptide mimics to facilitate their successful infection (Kim et al., 2018; Zhang et al., 2020; Mishra et al., 2023; Yimer et al., 2023). Investigating the functions of *CLE*-like genes of RKN will underscore the ecological significance of CLE signaling in plant-RKN interactions. Moreover, the ways which RKN and rhizobia infect host plants share similarities even though RKNs and rhizobia are parasites and symbionts, respectively (Costa et al., 2021). Advancing our knowledge about plant-rhizobia interactions may help to reveal the mechanisms of plant-RKN interactions.

### 4.2 Cyst nematodes

Parasitic cyst nematodes (CN; *Heterodera* and *Globodera* spp.) are obligate parasites to vascular plants including economically important crops (Jones et al., 2013). They penetrate their host roots and inject various effectors into the host procambial cells to establish their feeding site called syncytium in the root (Davis et al., 2008). Like AM fungi, CNs possess functional *CLE*-like genes in their genome as effectors. The prepropeptides of *Hterodera* species were similar to that of plants, while that of *Globodera* species possessed five CLE domains in addition to a signal peptide and a variable domain (Mitchum and Liu, 2022) (**Figure 1C**). Several studies in *H. glycines*, and *G. rostochiensis* showed that transcript of *GrCLE1* and *GrCLE4* was accumulated in dorsal gland secretory cells of the infective and parasitic juveniles, while immunolocalization experiments revealed that HgCLE peptides were localized in dorsal gland cells of parasitic juveniles (Wang et al., 2005, 2010; Bakhetia et al., 2007; Lu et al., 2009). Neither *HsCLEs* nor *HgCLEs* were expressed in pre-parasitic juveniles, implying that CN *CLE*-like genes act only in parasitic stages (Wang et al., 2010; Fosu-Nyarko et al., 2016). Heterologous expression of CN *CLE*-like genes (*HsCLE1*, *HsCLE2, HgCLE1*, *HgCLE2, GrCLE1*, and *GrCLE4*) that have high similarities of *AtCLE1*~*AtCLE7* mimicked *AtCLE* function; exogenous application of the 12 amino acids form of them inhibited primary root growth of *Arabidopsis* as well as AtCLE peptides (Wang et al., 2005, 2010, 2011; Lu et al., 2009; Replogle et al., 2011, 2013; Chen et al., 2015). Moreover, in planta-expressed *GrCLE1* was post-translationally processed, producing a 12 amino acids triarabinosylated GrCLE1 peptide which had high structural similarity to mature plant-CLE peptides, and this can directly bind to CLV2 of potato (Chen et al., 2015). The effect of *CLE*-like genes and peptides depends on the host receptor complexes such as AtCLV1 and AtCLV2 (Replogle et al., 2011, 2013; Chen et al., 2015; Guo et al., 2015, 2017), suggesting that the CN CLE-like peptides are recognized by receptors of the host plants. So, how do CN-CLE peptides injected into the host cells translocate to the apoplast to be perceived by the extracellular receptors? It has been shown that the variable domain of the CN CLE-like proteins has the function of a translocation signal that enables the CN CLE-like peptides to be delivered from the cytoplasm of syncytial cells to the apoplast through the host endoplasmic reticulum (Wang et al., 2010, 2021b).

The host receptor complexes also play a role in syncytium formation and CN fecundity. Mutants of AtCLV1, AtCLV2/AtCRN, AtRPK2, GmCLV2, and GmRPK2 exhibit defects of syncytium formation and low levels of CN fecundity (Replogle et al., 2011, 2013; Guo et al., 2015). In addition, exogenous treatment of HsCLEB, which is similar to TRACHEARY ELEMENT DIFFERENTIATION INHIBITORY FACTOR (AtTDIF), induces the expression of *WUSCHEL-RELATED HOMEOBOX 4* (*WOX4*) transcription factor, which promotes procambial cell proliferation, through TDIF-RECEPTOR (TDR) in roots, thereby facilitating syncytium formation (Guo et al., 2017). Gene expression profiles and genetic studies revealed that *HOMEOBOX GENE 8* (*AtHB8*), which controls vascular cell differentiation as well as *WOX4*, is a downstream component of CLE signaling during CN infection (Smetana et al., 2019; Liu and Mitchum, 2024). Also, direct binding of several CN CLE-like peptide/plant receptor pairs has been shown (Guo et al., 2011, 2015; Chen et al., 2015). Thus, it has been proposed that CNs co-opt the host developmental programs through CLAVATA pathways to support their infection (**Figure 2E**). However, whether CN CLE-like peptides injected into the host cells are processed during infection is not yet clear (Frei dit Frey and Favery, 2021).

### 4.3 Reniform nematode

Reniform nematodes (RN) also possess *CLE*-like genes. An RN, *Rotylenchulus reniformis*, is an obligate semi-endoparasite of more than 300 plant species. Three *RrCLE* genes were identified in the *R. reniformis* genome and consisted of a signal peptide at the N-terminus, a cryptic signal peptide within a variable domain, and a CLE domain at the C-terminus (Wubben et al., 2015) (**Figure 1C**). The *RrCLE* transcripts were detected in parasitic juveniles and localized in their dorsal gland. Another study in *G. max* infected by *R. reniformis* showed that several *GmCLE* genes were upregulated in the roots similarly to in the RKN-induced galls (Redding et al., 2018; Nakagami et al., 2023b). Therefore, RNs may use their CLE mimics and/or host-endogenous CLE peptides for their infection.

## 5 Involvement of CLE signaling in insect-induced gall formation

Some phytoparasitic insects induce gall formation on their host tissues where they acquire nutrients and are protected from enemies and environments (Favery et al., 2020). Phyloxera (*Daktulosphaira vitifoliae*), which parasitizes wild grapevine (*Vitis riparia*), induces the formation of flower-like gall on the host leaf. A transcriptomic analysis has found that the CLE signaling pathway in regulating cambial cell homeostasis is activated in *D. vitifoliae*-inducing galls (Schultz et al., 2019). For instance, *VrCLE44*, *VrTDR*, and *VrWOX4* are up-regulated in insect-inducing galls, as well as in RKN- and CN-inducing galls/syncytia (Yamaguchi et al., 2017; Schultz et al., 2019). Gall-forming insects may therefore use the mechanism for the cambial cell maintenance of their hosts.

## 6 Involvement of CLE signaling in plant-pathogen interactions

**Figure 3:**
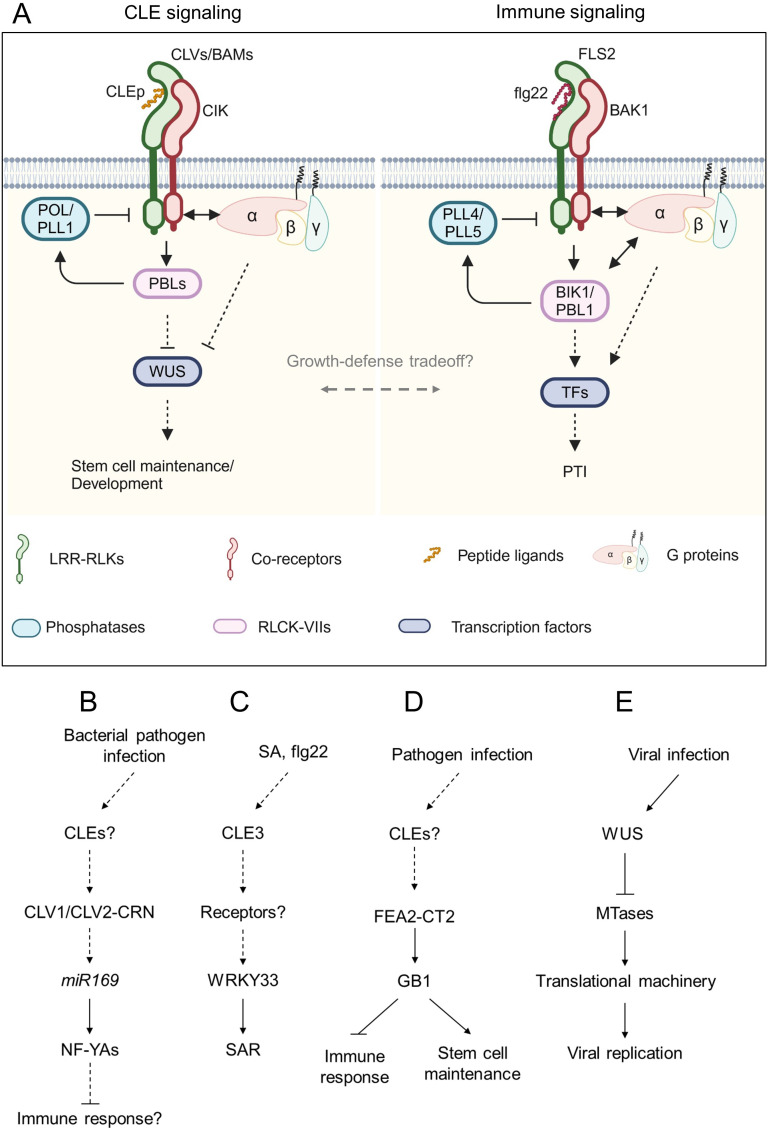
Schematic model of CLE signaling involved in immune response. **(A)** Representative model of the conserved signaling pathway between CLE signaling and immune signaling. Peptide ligands are recognized by leucine-rich repeat receptor-like kinases (LRR-RLKs) with their co-receptors. The signals are transduced via receptor-like cytoplasmic kinases (RLCKs) and G proteins and dampened by phosphatases to regulate downstream transcription factors. **(B-E)** Schematic flowcharts of CLE signaling involving plant-pathogen interactions. **(B-D)** Involvement of CLE signaling in plant-bacterial pathogen interactions in Arabidopsis **(B, C)** and maize **(D)**. **(E)** Involvement of WUS in plant-virus interaction in Arabidopsis. Dashed lines indicate putative or indirect pathways. Figure adapted from images created with BioRender.com.

Plants employ RKs and RLPs as pattern recognition receptors (PRRs) to recognize pathogen-associated molecular patterns (PAMPs), damage-associated molecular patterns (DAMPs), and phytocytokines (Zhang et al., 2023b). PRRs induce pattern-triggered immunity (PTI) by recognizing PAMPs. One of the best-studied PRRs is FLAGELLIN SENSITIVE 2 (FLS2) which recognizes bacterial flagellin with the co-receptor BAK1 via a 22 amino acids epitope (flg22) (DeFalco and Zipfel, 2021). Emerging evidence suggests that CLE signaling is involved in plant immune responses and signaling by CLEs-CLV1/BAMs in development and flg22-FLS2 in immunity uses similar downstream factors (DeFalco et al., 2022) (**Figure 3A**). Here we discuss the current knowledge on the contribution of CLE signaling to plant immunity.

AtCLV1 and AtCLV2 participate in susceptibility to bacterial, fungal, and oomycete pathogens (**Figure 3B**). The *clv1* and *clv2* mutants exhibited increased resistance against the bacterial pathogen *Ralstonia solanacearum* and the oomycete pathogen *Hyaloperonospora arabidopsidis* compared to the wild-type plant (Hanemian et al., 2016). Conversely, *clv1* was more susceptible to the bacterial pathogen *Pseudomonas syringae* pv. *tomato* DC3000 and the fungal pathogens *Plectosphaerella cucumerina* and *Botrytis cinerea*. Multiple *NF-YA* genes were upregulated in *clv1* and *clv2* mutants infected with *R. solanacearum* in an *miR169*-dependent manner. The overexpression of *miR169* that suppresses the *NF-YA* genes facilitated susceptibility of *clv1* and *clv2* to *R. solanacearum* (Sorin et al., 2014; Hanemian et al., 2016). In contrast to the more susceptible phenotype of *clv1* to *B. cinerea*, the mutant lacking AtACR4, which recognizes CLE40p coordinately with AtCLV1, was more resistant to this fungal pathogen (E-Zereen and Ingram, 2012; Czyzewicz et al., 2016). On the one hand, work by Lee et al. (2011) has proposed that AtCLV3 peptide is recognized by AtFLS2 leading to activation of immune response in the SAM. On the other hand, work by Segonzac et al. (2012) has provided experimental evidence that AtFLS2 does not recognize AtCLV3 peptide and that the immunity of the SAM to DC3000 is independent of AtCLV3 peptide perception. Another study focused on gene expression analyses of *AtCLE* genes has revealed that *AtCLE3* expression is induced in the roots by exogenous treatments of flg22, Pep2, which is a 23 amino acids peptide known as a DAMP, and the defense phytohormone salicylic acid (SA) (Ma et al., 2022). Root-expressing *AtCLE3* is required for upregulation of shoot-expressed *WRKY33* that contributes to systemic acquired resistance (Wang et al., 2018; Ma et al., 2022), suggesting that the AtCLE3 peptide may act as a systemic signal to transduce SA signaling from roots to shoots (**Figure 3C**). However, it has not been tested yet whether AtCLE3 peptide contributes to immune response, particularly affecting pathogen growth in planta. Therefore, careful investigation of whether and how the CLE signaling is involved in plant immunity is necessary.

Recent studies have also provided evidence that downstream components of the CLE-receptor modules are involved in plant immunity. Genetic studies have shown that Arabidopsis G proteins AtAGB1 (Gβ), AtAGG1, and AtAGG2 (Gγ) are required for FLS2-mediated immune response (Liu et al., 2013). Furthermore, AtAGB1 and EXTRA-LARGE GTP BINDING PROTEIN 2 (AtXLG2), a noncanonical Gα, directly interact with FLS2 and BOTRYTIS-INDUCED KINASE 1 (BIK1), conferring stability on the receptor complex (Liang et al., 2016). AtAGB1 also interacts with AtRPK2, which maintains meristem activity in the SAM (Ishida et al., 2014), suggesting that G proteins control both meristem development and immune response. A recent study found that the *Zea mays Gβ subunit 1* (*ZmGB1*) mutants showed seedling-lethal phenotype due to autoimmunity (Wu et al., 2020b). A viable *ZmGB1* mutant fea*183 was identified from ethyl methanesulfonate-mutagenesis screen and showed striking inflorescence defects, reminiscent of mutants lacking ZmCT2 (Gα) (Bommert et al., 2013; Wu et al., 2020b). *ZmFEA2*, an ortholog of *AtCLV2*, and *ZmCT2* were epistatic to *ZmGB1*, suggesting that the CLE-CLV-G protein signaling circuit may balance the tradeoff between growth and defense (**Figure 3D**). The SAM-specific transcription factor WUS protects the SAM from infection by cucumber mosaic virus (CMV) (Wu et al., 2020a; Lopes et al., 2021). WUS inhibits transcript levels of *S*-adenosyl-Lmethionine-dependent methyltransferases (MTases), which are involved in rRNA processing and ribosome stability, by responding to CMV infection, thereby sabotaging CMV replication and its invasion ability to the SAM (Wu et al., 2020a) (**Figure 3E**).

Apart from the CLE signaling pathway, plant endogenous signaling peptides play roles in plant immunity, abiotic stress response, and growth regulation (Fitrianti et al., 2022; Liu et al., 2022; Rzemieniewski and Stegmann, 2022; Rzemieniewski et al., 2022; Taleski et al., 2024). In addition, a recent study has revealed that a RALF-FERONIA signaling module that modulates the formation of FLS2-BAK1 receptor complex affects the rhizosphere microbiome (Song et al., 2021). This suggests that peptide-receptor modules are central regulators of diverse aspects of plant physiology, and their roles in immunity, development, and beyond are mechanistically coupled. Yet, how the CLE signaling harmonizes multiple aspects of plant immunity, development, and physiology is still elusive.

## 7 Conclusion

Peptide ligand and its receptor pairs play important roles in plant development and plant-microbe interactions. The CLE gene family is one of the largest among those that encode peptide ligands, and they are conserved across green algae to higher plants (Olsson et al., 2019). Plants have evolved the CLE signaling to develop more complex multicellular bodies and be adapted to environmental nutritional status that is constantly changing. Both developing *de novo* organs induced by plant-associated microbes and coping with the nutritional status of their host are pivotal for microbial colonization. Thus, plant-associated microbes may utilize the host-CLE signaling to achieve those.

Secreted peptides are perceived by distinct receptors in the apoplast, in consequence, their signals are transduced into the cell. In this process, post-translational modifications to form mature peptides are required. However, the mature form of most CLE peptides involved in plant-microbe interactions remains uncharacterized. In addition, most of the knowledge about peptide-receptor pairs has been brought by genetic studies, it is still largely unknown whether distinct peptides bind their cognate receptors. Interestingly, it has been shown that AI-based prediction to identify peptide ligand and receptor pairs is a powerful tool for structure-function analysis of peptide-receptor pairs (Snoeck et al., 2024). Applying this in silico method to CLE peptides and their postulated receptors holds the potential to identify bona fide peptide-receptor pairs more and distinguish functions of different peptides.

While there are increasing studies on plant-microbe interactions, such efforts still lag behind those on plant development and physiology due to more complex phenomena and technical obstacles. Recently, several studies have tackled unraveling molecular mechanisms underlying plant-microbe interactions by using the co-transcriptomics of plants inoculated with microbes and the microbes in planta (Roux et al., 2014; Hacquard et al., 2016; Nobori et al., 2018, 2022; Sauviac et al., 2022; Siddique et al., 2022; Zhang et al., 2023a), spacial transcriptomics (Frank et al., 2023; Nobori et al., 2023; Schnabel et al., 2023; Tang et al., 2023; Verbon et al., 2023; Zhu et al., 2023), and the technique that is combined with both (Serrano et al., 2024a, 2024b), which will identify yet unknown upstream and downstream components of the CLE-receptor modules, and accelerate our understanding of ecological implications of the plant-microbe interactions.
